# In vivo emergence of beige-like fat in chickens as physiological adaptation to cold environments

**DOI:** 10.1007/s00726-021-02953-5

**Published:** 2021-02-17

**Authors:** Rina Sotome, Akira Hirasawa, Motoi Kikusato, Taku Amo, Kyohei Furukawa, Anna Kuriyagawa, Kouichi Watanabe, Anne Collin, Hitoshi Shirakawa, Ryota Hirakawa, Yuta Tanitaka, Hideki Takahashi, Guoyao Wu, Tomonori Nochi, Tsuyoshi Shimmura, Craig H. Warden, Masaaki Toyomizu

**Affiliations:** 1 Animal Nutrition, Life Sciences, Graduate School of Agricultural Science, Tohoku University, Sendai, 980-8572 Japan; 2 Department of Genomic Drug Discovery Science, Graduate School of Pharmaceutical Sciences, Kyoto University, Kyoto, 606-8501 Japan; 3 International Education and Research Center for Food and Agricultural Immunology, Graduate School of Agricultural Science, Tohoku University, Sendai, 980-8572 Japan; 4 Department of Applied Chemistry, National Defense Academy, 1-10-20 Hashirimizu, Yokosuka, 239-8686 Japan; 5 Functional Morphology, Life Sciences, Graduate School of Agricultural Science, Tohoku University, Sendai, 980-8572 Japan; 6 INRAE, Université de Tours, BOA, 37380 Nouzilly, France; 7 Nutrition, Graduate School of Agricultural Science, Tohoku University, Sendai, 980-8572 Japan; 8 Plant Pathology, Life Sciences, Graduate School of Agricultural Science, Tohoku University, Sendai, 980-8572 Japan; 9 Department of Animal Science, Texas A & M University, 2471 TAMU, College Station, TX 77843-2471 USA; 10 Graduate School of Agricultural Science, Tokyo University of Agriculture and Technology, Fuchu, 183-8509 Japan; 11 Department of Pediatrics, Section of Neurobiology, Physiology and Behavior and Rowe Program in Genetics, University of California, Davis, CA 95616 USA

**Keywords:** Avian models, avUCP, Beige fat, Thermogenesis, Cold adaptation

## Abstract

While it has been hypothesized that brown adipocytes responsible for mammalian thermogenesis are absent in birds, the existence of beige fat has yet to be studied directly. The present study tests the hypothesis that beige fat emerges in birds as a mechanism of physiological adaptation to cold environments. Subcutaneous neck adipose tissue from cold-acclimated or triiodothyronine (T_3_)-treated chickens exhibited increases in the expression of avian uncoupling protein (*avUCP, an ortholog of mammalian UCP2 and UCP3*) gene and some known mammalian beige adipocyte-specific markers. Morphological characteristics of white adipose tissues of treated chickens showed increased numbers of both small and larger clusters of multilocular fat cells within the tissues. Increases in protein levels of avUCP and mitochondrial marker protein, voltage-dependent anion channel, and immunohistochemical analysis for subcutaneous neck fat revealed the presence of potentially thermogenic mitochondria-rich cells. This is the first evidence that the capacity for thermogenesis may be acquired by differentiating adipose tissue into beige-like fat for maintaining temperature homeostasis in the subcutaneous fat ‘neck warmer’ in chickens exposed to a cold environment.

**Supplementary Information:**

The online version contains supplementary material available at 10.1007/s00726-021-02953-5.

## Introduction

How different species of organisms adapt to environmental temperature is central to understanding mechanisms for animal resilience in response to climate change. In mammals, two types of thermogenic fat cells, brown adipocytes and beige adipocytes, have been identified and play key roles in the regulation of systemic energy homeostasis. Brown adipocytes contain an abundance of mitochondria and small multilocular lipid droplets. The main protein responsible for non-shivering thermogenesis (NST) is uncoupling protein 1 (UCP1), which is embedded in the mitochondrial inner membrane and dissipates the proton gradient to generate heat (Cinti et al. 1989; Nicholls et al. 1978). Similarly, brown-like adipocytes (beige adipocytes or brite [brown-in-white] adipocytes) also have a multilocular morphology and express UCP1, but can be found in white adipose depots only when animals are exposed to cold or other inducers (Paulo and Wang 2019; Himms-Hagen et al. 2000; Vitali et al. 2012; Young et al. 1984; Kajimura et al. 2015). Regarding typical similarities and differences between brown adipocytes and beige adipocytes in mammals, cell death-inducing DNA fragmentation factor like effector a (CIDEA) is expressed in both brown and beige adipocytes (Ikeda et al. 2018; Petrovic et al. 2010; Sharp et al. 2012; Wu et al. 2012), and T-box transcription factor 1 (TBX1) and transmembrane protein 26 (TMEM26) (Harms and Seale 2013; Ikeda et al. 2018; Wu et al. 2012), are specifically expressed in beige adipocytes.

For birds, morphological data suggest that there is no BAT, but BAT-resembling WAT (Barre et al. 1986a; Oliphant 1983). However, this WAT lacks the metabolic capacity characteristic of BAT and has neither sympathetic innervation nor expression of UCP1, which is the key protein for BAT-type thermogenesis (Saarela et al. 1989, 1991). Based on these observations, Hohtola (2002) hypothesized that there are no adipose tissues, such as BAT or beige fat, which can participate in thermoregulatory heat production in birds. Just before proposing his hypothesis, Raimbault et al. (2001) and Vianna et al. (2001) made the discovery of a novel uncoupling protein (avian UCP) in skeletal muscle of birds. The expression of avUCP in skeletal muscle in chickens was elevated by exposure to cold-(Collin et al. 2003a; Toyomizu et al. 2002; Ueda et al. 2005) and T_3_-(Collin et al. 2003b) treatment, both of which are known to be typical stimuli for the emergence of beige adipocytes in mammals. However, no studies have examined expression of avUCP in beige fat tissues to confirm its existence in birds. Studies investigating the emergence of beige adipocytes are of particular interest because it is unclear whether skeletal muscle is the only important source of cold-induced non-shivering thermogenesis in birds. Consequently, in our study, we provide evidence for the in vivo emergence of beige-like fat within neck subcutaneous fat in chickens by (1) examining the effects of cold acclimation and T_3_ treatment on the expression of the *avUCP* gene and other marker genes known to be enriched in mammalian beige fat, as well as on the morphological characteristics of neck subcutaneous and abdominal fat adipose tissues of chickens; (2) determining the mRNA and protein levels of avUCP and the protein abundance of the mitochondrial marker, voltage-dependent anion channel (VDAC); and (3) analyzing mitochondria in multilocular fat cells from the neck subcutaneous fat of T_3_-treated chickens using immunohistochemistry.

## Materials and methods

### Ethics statement

The Animal Care and Use Committee of the Graduate School of Agricultural Science, Tohoku University, approved all procedures and every effort was made to minimize pain or discomfort to all animals used in the study.

### Animals and experimental design

One-day-old male white leghorn chicks (Julia) were obtained from a commercial hatchery (I-Hiyoko, Co. Ltd, Niigata, Japan). The chicks were housed in electrically heated batteries and provided with water and a commercial starter diet ad libitum. Fourteen-day-old chickens that were to be used for experiments were selected from a twofold larger population to obtain uniform body weights and kept in wire-bottomed cages under conditions of controlled temperature (24 ± 1 °C) and continuous light for 6–7 days.

In the first series of experiments, twenty-four 21-day-old chickens (235 ± 11 g), after a 3-day adaptation period for individual cages were used for confirmation of increased expression of *avUCP* and beige adipose tissue markers, as well as the emergence of multilocular fat cells in cold-exposed or T_3_-treated chickens. The chickens were randomly divided into the following three groups (*n* = 8 per group): a ‘control’ group, fed a commercial diet under a thermoneutral environment (24 ± 1 °C); a ‘T_3_’ group, fed a commercial diet containing thyroid hormone (0.6 mg/kg of diet: T3, #T2877, Sigma-Aldrich, St. Louis, MO, USA) under thermoneutral conditions; and a ‘cold exposure’ group, fed a commercial diet in a cold environment (4 ± 1 °C) and then maintained for 10 days. Individual body weight and feed intake were recorded.

In a second series of experiments, twelve 20-day-old chickens (219 ± 6 g) after a 3-day adaptation period were used to clarify the presence of beige adipocyte clusters, constituting small adipocytes in the mitochondria-rich tissue of T_3_-treated chickens. Six chickens were fed a commercial diet (‘control’ group), while the remainder were fed a commercial diet supplemented with T_3_ (0.6 mg/kg of diet) under thermoneutral conditions (‘T_3_’ group), and then maintained for 9 days.

The dose of T_3_ was based on preliminary trial (unpublished data) and previous study (Collin et al. 2003b). All chickens had free access to food and water. After they were decapitated, neck subcutaneous and abdominal fats were quickly excised. They were then weighed, frozen, ground to a powder in liquid nitrogen, and stored at − 80 °C until used for protein analyses and extraction of total RNA. For analysis of fat tissue morphology, a sample of fat excised from each chicken was fixed in 10% formalin neutral buffer solution.

### Quantitative real-time RT-PCR

Total RNA was extracted and cDNA was synthesized as previously described (Kikusato et al. 2015), with minor modifications. A quantitative RT-PCR analysis was performed using a CFX Connect™ system (Bio-Rad Laboratories, Hercules, CA, USA) for measuring the expression levels of *avUCP*, *CIDEA* (Ikeda et al. 2018; Petrovic et al. 2010; Sharp et al. 2012; Wu et al. 2012), *TBX1*, *TMEM26* (Harms and Seale 2013; Ikeda et al. 2018; Wu et al. 2012), *CAR4* (carbonic anhydrase 4) (Sharp et al. 2012), *SLC27A1* (solute carrier family 27 member 1, fatty acid transporter) (Sharp et al. 2012; Wu et al. 2012), *CD137* (Wu et al. 2012; Harms and Seale 2013), *EAR2* (NR2F6: nuclear receptor subfamily 2 group F member 6) (Wu et al. 2012), and *CPT1b* (carnitine palmitoyltransferase 1b) (Sharp et al. 2012). The results are presented as ratios of mRNA levels of target molecules to 18S ribosomal RNA (18S). Primer sets used to amplify each gene are listed in Table S1.

### Hematoxylin and eosin (H&E) staining

Adipose tissue was collected from each chicken and fixed in 10% formalin neutral buffer solution for 24 h at room temperature (25 °C). The tissues were dehydrated with 70%, 80%, 90%, 95%, 100%, and 100% (v/v) ethanol, treated with xylene, and embedded in paraffin. Tissue sections (4 µm thick) were prepared using a microtome and dehydrated with a graded ethanol series and stained with hematoxylin and eosin (H&E) to address the cell structure under a microscope.

To measure adipocyte size distribution, H&E-stained tissue images were subjected to grayscale conversion, and further binarization was carried out by setting the pixel existing in the cell area to 1 and other pixels to 0. Regions in which binarization could not be appropriately performed, as determined by visual inspection, were either deleted or corrected manually. The maximum area where the pixels of the cell area are connected to each other is judged as one cell and the pixel number is considered as the cross sectional area of the cell. To remove noise, cells with less than 10 pixels (an extremely small area) were removed. Image processing was performed using R script (https://www.r-project.org/) and ImageJ (v1.29x, National Institute of Health, Bethesda, MD, USA).

For the detection of small cell clusters, cells with an area larger than the threshold values of (250, 500, and 1000) pixels, were removed from the binary image at the time of measurement of the distribution of the cell area, and a binarized image was created by extracting only small cells. After applying a Gaussian filter to the image, binarization was again measured. Isolated cells were left intact and clusters of small cells were classified as connected regions by the blurred boundaries. The connected region was judged to be a cluster of small cells and measurements were carried out. Calculations were performed with the NumPy library, matplotlib, and scikit-image in Python (https://www.python.org/).

### Immunohistochemistry

Histological analyses were carried out as described previously (Niimi et al. 2018; Zhu et al. 2017) with some modifications. The sections were washed with water after deparaffinization in xylene and dehydration in ethanol. The sections were incubated in 3% H_2_O_2_ diluted with phosphate buffered saline (PBS) for 15 min to remove endogenous peroxidase activity and incubated in 10 mM citrate buffer for 60 min at 90 °C for antigen retrieval. After three 5-min PBS washes, the sections were blocked in 10% goat serum (#426041, Nichirei Bioscience, Tokyo, Japan) for 10 min and incubated with a polyclonal rabbit anti-VDAC primary antibody (ab15895, Abcam, Cambridge, UK) at a dilution of 1:3000 overnight at 4 °C. Normal rabbit IgG (#2729, Cell Signaling, Beverly, MA, USA) diluted to the same concentration as a primary antibody was used as the negative control. After being washed three times for 5 min in PBS, the sections were incubated in Histofine Simple Stain Max PO (#414181, Nichirei Bioscience) as a secondary antibody at room temperature for 30 min. After three 5-min washes in PBS, the signals were developed using a diaminobenzidine (DAB) substrate solution kit (#425011, Nichirei Bioscience) and counterstained with hematoxylin.

### Western blotting

To measure VDAC/Porin and avUCP protein levels, western blotting was conducted as previously described, with minor modifications (Kikusato and Toyomizu 2013). The frozen tissues were solubilized/sonicated with radioimmunoprecipitation assay (RIPA) buffer. Lysates were mixed with the same amount of sodium dodecyl sulfate (SDS) buffer (containing 0.125 M Tris–HCl [pH 6.8], 4% SDS, 10% glycerol, 0.002% phenol red, and 0.1 M dithiothreitol), and then boiled for 5 min. The samples were loaded onto 12% polyacrylamide gels (Mini-PROTEAN® TGX™ Gels, Bio-Rad). The electrophoresed proteins were transferred to a polyvinylidene difluoride (PVDF) membrane (0.2 µm) using a semi-dry transfer apparatus (Trans-Blot® Turbo™, Bio-Rad) according to the manufacturer’s instructions. The membranes were blocked with Tris-buffered saline (TBS) containing 5% (*w/v*) skimmed milk and 0.1% (*v/v*) Tween 20 for 1 h at room temperature. The membranes were incubated overnight at 4 °C with rabbit anti-VDAC (ab15895, Abcam) or rabbit anti-avUCP polyclonal antibodies diluted 1:3000 and 1:100, respectively. Anti-avUCP antibody was obtained from rabbits by injection of a peptide (MVGLKPPEVPPTAAVK) coupled with the KLH protein (EFS, Etablissement français du sang, Nantes, France). After being washed, the membranes were incubated with a horseradish peroxidase (HRP)-conjugated anti-rabbit IgG secondary antibody (7074S, Cell Signaling Technology, Beverly, MA, USA) diluted 1:10,000 in blocking buffer at room temperature for 1 h. After being washed, the signal was developed with a chemiluminescent substrate solution (Chemi-Lumi One Super, Nacalai Tesque Inc., Kyoto, Japan) for 1 min. Immunoreactive proteins on the membranes were imaged using a VersaDoc Model 5000 (Bio-Rad). Protein sizes were estimated using a MagicMark™ Western protein standard (#LC5602, Thermo Fisher Scientific, Waltham, MA, USA). The VDAC/Porin protein content was quantified based on standard curves obtained from serial dilutions of recombinant human VDAC1/Porin (ab132481, Abcam) on the same membrane. Additionally, membranes were stained with Ponceau-S (#SP-4030, Aproscience, Tokushima, Japan) according to the manufacturer’s instructions to confirm similarities of protein transfer efficiency among the loading western blot samples.

### Statistics

Statistical analyses were performed using BellCurve for Excel 2015 software (Social Survey Research Information Co., Ltd. Tokyo, Japan). Differences between control, T_3_- and cold-treated groups and between control and T_3_-treated groups were assessed using a Shirley-Williams test and a Student’s *t*-test for unpaired data, respectively. Differences in data with *p* < 0.05 are considered statistically significant. Pearson correlation coefficients were calculated between *avUCP* and *PGC-1α* gene levels.

## Results

### Body weight, food intake, and adipose tissue weight

**Fig. 1:**
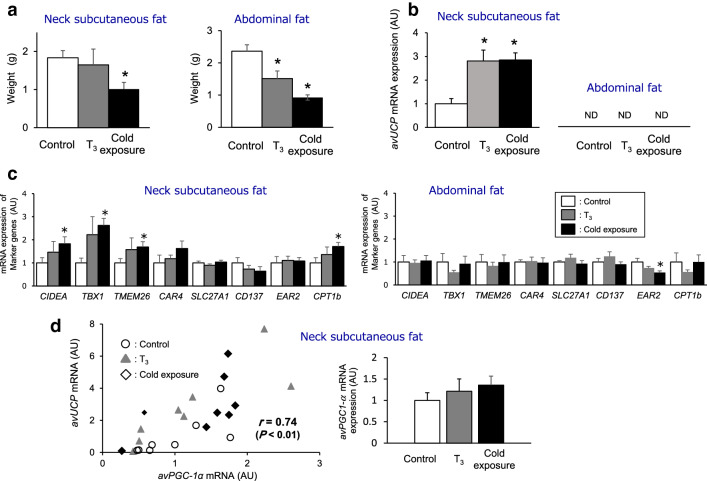
Increased mRNA expression levels of avian *UCP* (*avUCP*) and of beige adipose tissue markers in chickens exposed to 4 °C or fed a T_3_-supplemented diet. **a** Weights of neck subcutaneous fat (left) and abdominal fat (right) of control, T_3_-treated and cold-exposed chickens. Values are means ± S.E., *n* = 8 chickens in each group. **b** mRNA levels of *avUCP* in neck subcutaneous fat (left) and abdominal fat (right) of control, T_3_-treated and cold-exposed chickens. Values are means ± S.E., *n* = 8 chickens in each group. **c** mRNA levels of markers known to be enriched in mammalian beige adipocytes in neck subcutaneous fat (left) and abdominal fat (right) of control, T_3_-treated and cold-exposed chickens. Values are means ± S.E., *n* = 8 chickens for *CIDEA*, *TBX1* and *TMEM26* in each group; *n* = 5–8 chickens for *CAR4*, *SLC27A1*, *CD137*, *EAR2* and *CPT1b* in each group. **d** Dependency of *avUCP* expression on *avPGC-1α* in neck subcutaneous fat of control, T_3_-treated, and cold-exposed chickens (*n* = 24) and effects of T_3_- and cold-treatments on mRNA levels of *avPGC-1α*. (*n* = 8 chickens in each group). All data for mRNA levels are shown as fold changes relative to control values. Quantitative real-time RT-PCR was used to quantify mRNA levels, and the results were normalized to 18S mRNA levels. ND means not appropriately detected for too low expression. Value in panel D represents Pearson correlation coefficient for the relationship between *avUCP* and *PGC-1α* gene levels. Differences between control, T_3_- and cold-treated groups were assessed using the Shirley-Williams test. *p < 0.05 compared to the control group

Compared with the control group, body weight gain was marginally lower in T_3_-treated chickens and significantly lower in cold-acclimated chickens (control, 147 ± 13 g; T_3_-treated, 135 ± 15 g; cold-acclimated, 81 ± 13 g: *p* < 0.05 versus control). T_3_-treated chickens ate significantly less feed than the control, whereas cold-acclimated chickens ate significantly more (control, 831 ± 16 g; T_3_-treated, 770 ± 24 g: *p* < 0.05 versus control; cold-acclimated, 1009 ± 29 g: *p* < 0.05 versus control). Consequently, and as expected, feed efficiency, which is calculated as the ratio of body weight gain to feed intake, decreased in cold-acclimated animals, but not in T_3_-treated chickens (control, 0.177 ± 0.004; T_3_-treated, 0.175 ± 0.005; cold-acclimated, 0.080 ± 0.005: *p* < 0.05 versus control), implying that cold acclimation induced greater whole-body heat production. Neck subcutaneous fat weight was significantly lower in cold-acclimated animals than in control chickens, but there was no significant difference between T_3_-treated and control chickens, whereas abdominal fat weight was significantly lower in T_3_-treated and cold-acclimated chickens than in control chickens (Fig. 1a).

### mRNA expression of *avUCP* and mammalian beige adipocyte-related genes

To determine the mRNA expression levels of typical gene markers known to be enriched in mammalian beige adipocytes, the mRNA levels of *avUCP*, *CIDEA*, *TBX1*, *TMEM26, CAR4, SLC27A1, CD137, EAR2,* and *CPT1b* in the neck subcutaneous and abdominal fats of T_3_-treated and cold-exposed chickens were analyzed by quantitative real-time RT-PCR (Fig. 1b, c). There was a statistically significant increase in *avUCP* mRNA levels in the neck subcutaneous fat of T_3_-treated and cold-acclimated chickens compared with control chickens. In contrast, in the abdominal fat *avUCP* mRNA was not detected in control, T_3_-treated or cold-acclimated chickens (Fig. 1b). In neck subcutaneous fat, the mRNA expression levels of *CIDEA*, *TBX1*, *TMEM26* and *CPT1b* were significantly higher, though the level of CAR4 was slightly higher, in the cold-acclimated group than in the control group, while in the T_3_-treated group, those levels were intermediate between control and cold-acclimated groups. The levels of *SLC27A1, CD137,* or *EAR2* mRNA did not increase in both treatments (Fig. 1c). In contrast, no differences were observed in abdominal fat *CIDEA*, *TBX1*, *TMEM26, CAR4, SLC27A1, CD137, EAR2,* and *CPT1b* mRNA expression levels except for *EAR2* mRNA whose level was decreased by both treatments. To clarify whether avian peroxisome proliferator-activated receptor γ coactivator-1α (*PPARGC1A*, *avPGC-1α*) is associated with *avUCP* expression in neck subcutaneous fat, the dependency of *avUCP* expression on avPGC-1α was investigated. The results showed a positive correlation between *avUCP* and *avPGC-1α* mRNA levels of control, T_3_-treated and cold-exposed chickens despite the observation that *avPGC-1α* was not significantly increased by either treatment (Fig. 1d).

### Morphological characteristics of neck subcutaneous fat and abdominal fat stained with hematoxylin and eosin

**Fig. 2:**
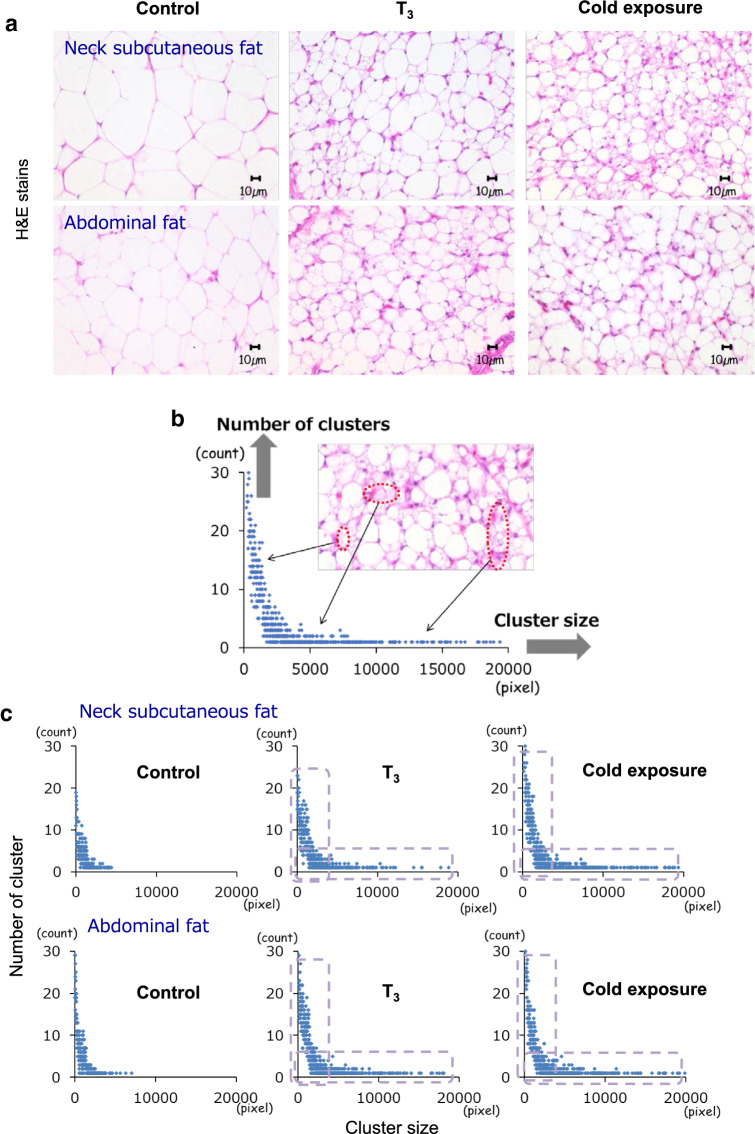
Emergence of multilocular fat cells in chickens exposed to cold and fed a T_3_-supplemented diet. **a** H&E stains showing multilocular fat cells within the neck subcutaneous fat (upper) and abdominal fat (lower) depots in control, T_3_-treated and cold-acclimated chickens. **b** Process for the quantitative evaluation of multilocular adipocytes. Fat depots containing small adipocytes were regarded as one cluster, such as large, medium, or small-sized cluster. **c** Scatter plots displaying data points of the number of clusters on the *y*-axis versus the cluster size on the *x*-axis within neck subcutaneous fat (upper) and abdominal fat (lower) tissues of control, T_3_-treated and cold-acclimated chickens

Hematoxylin and eosin (H&E) staining was carried out to investigate the emergence of the beige adipocytes by cold and T_3_ treatment. Islets of multilocular fat cells were observed within the neck subcutaneous fat and abdominal fat depots of T_3_-treated and cold-acclimated chickens though there were few multilocular fat cells in control chickens (Fig. 2a): namely, fat cells became more multilocular in T_3_-treated and cold-acclimated chickens, i.e., the cells were smaller. These morphological characteristics are very similar to those observed for stimuli-induced mammalian beige fat (Sidossis and Kajimura 2015). For quantitative evaluation of multilocular adipocytes, each fat depot consisting of small adipocytes was regarded as one cluster, and digital imaging was used to measure the number of clusters relative to each cluster size within the WAT depots (Fig. 2b). Scatter plots were constructed as shown in Fig. 2c. Compared with the control group, cold- or T_3_-treated chickens exhibited increases in the numbers of both small and larger clusters, showing an increased number of multilocular fat cell clusters within neck subcutaneous fat and abdominal fat, and providing evidence for the emergence of multilocular fat cells as a result of T_3_ and cold stimulation.

### mRNA and protein levels of avUCP in tissue and mitochondrial VDAC protein content

**Fig. 3:**
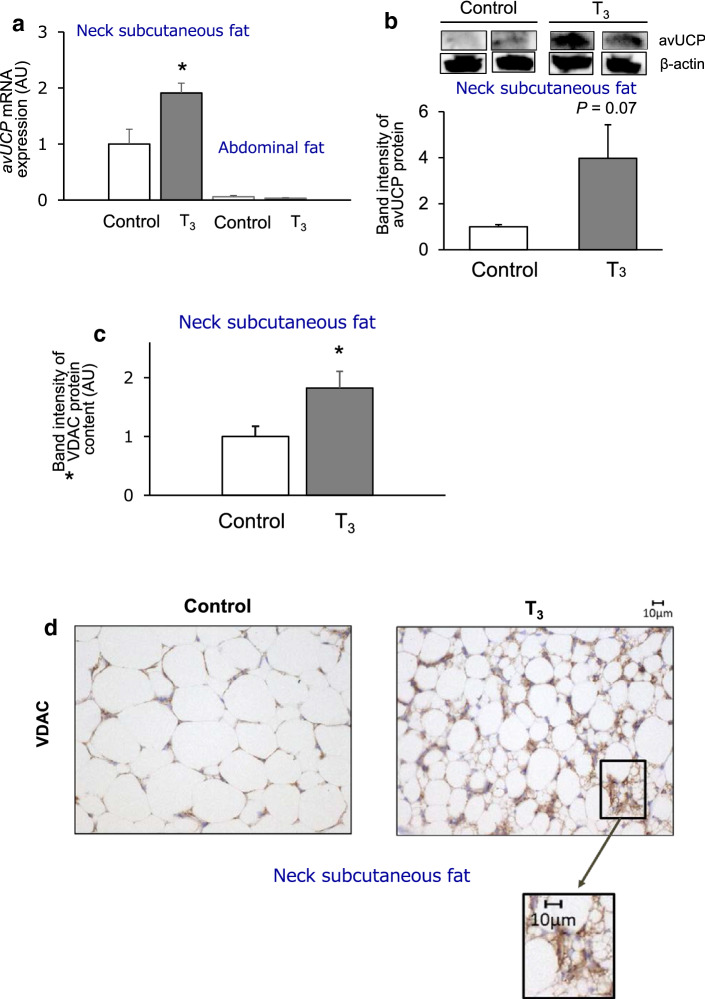
Clusters of beige-like adipocytes densely emerge in a mitochondria-rich cell of neck subcutaneous fat in T_3_-treated chickens. **a** mRNA levels of *avUCP* in neck subcutaneous fat and abdominal fat tissues of control and T_3_-treated chickens. Quantitative real-time RT-PCR was used to quantify mRNA levels, and the results were normalized to 18S rRNA levels. Values are means ± S.E., *n* = 5–6 chickens in each group. **b** Protein levels of avUCP in neck subcutaneous fat of control and T_3_-treated chickens. avUCP protein levels were assessed by Western blot of tissue protein (80 μg) using an anti-avUCP antibody. Band intensities of avUCP shown as semi-quantified by densitometric tracing. Values are means ± S.E., *n* = 5 chickens in each group. Uncropped data and observed band size are depicted in Supplement A, Supporting Information. **c** VDAC protein content in neck subcutaneous fat of control and T_3_-treated chickens. VDAC protein levels were assessed by Western blot analysis of tissue protein (10 μg) using an anti-VDAC antibody. Band intensities of VDAC shown as semi-quantified by densitometric tracing. Values are means ± S.E., *n* = 6 chickens in each group. Uncropped data are depicted in Supplement B, Supporting Information. **d** Immunohistochemical analysis using anti-VDAC antibody for neck subcutaneous fat of control and T_3_-treated chickens. Data for mRNA and protein levels of avUCP or VDAC protein are shown as fold changes relative to control values for the neck subcutaneous fat. Differences between control and T_3_-treated chickens were assessed using the Student’s t-test for unpaired data. *p < 0.05 compared to the control group

In the second series of experiments, mRNA and protein levels of avUCP and the mitochondrial protein VDAC were examined to characterize the neck subcutaneous fat of T_3_-treated chickens. The level of *avUCP* mRNA expression significantly increased in the T_3_-treated group compared with the control group though the levels were very low in the abdominal fat of both groups (Fig. 3a). A positive correlation between *avUCP* and *avPGC-1α* mRNA levels of control and T_3_-treated chickens was observed (Fig. S1). These results were in agreement with findings obtained from the first series of experiments. The avUCP protein content also tended to increase with T_3_ treatment (Fig. 3b). The protein expression level of VDAC, which is a mitochondrial marker protein, expressed per tissue protein, significantly increased in the neck subcutaneous fat of T_3_-treated chickens (Fig. 3c), which means that an enhancement of mitochondrial biogenesis follows T_3_ stimulation in the neck subcutaneous fat. In contrast, the levels of *avPGC-1α* mRNA and the VDAC protein in abdominal fat were not significantly different between the groups (Fig S2 and S3).

### Immunohistochemistry of neck subcutaneous fat from T_3_-treated chickens

Morphological and immunohistochemical characterizations of the neck subcutaneous fat of T_3_-treated chickens were carried out using an anti-VDAC antibody. The neck subcutaneous fat of T_3_-treated chickens presented many types of clusters that were positive for VDAC, corresponding to multilocular fat cells (Fig. 3d). The abdominal fat exhibited weaker VDAC signal in both groups compared with the neck subcutaneous fat, and there was no apparent difference in the signal between groups (Fig. S4). Therefore, it could be concluded that the dense clusters of beige-like adipocytes were present in the mitochondria-rich neck subcutaneous fat tissue of T_3_-treated chickens.

## Discussion

Brown adipocytes in mammals are distinguished from the more common white fat adipocytes by having numerous small lipid droplets, elevated numbers of mitochondria, and mitochondrial expression of UCP1, the uncoupler of oxidative phosphorylation responsible for non-shivering thermogenesis (Mezentseva et al. 2008). Although the thermogenic BAT has not been described in birds, Mezentseva et al. (2008) identified in vitro inductive conditions for avian brown adipocyte-like cells and demonstrated that mesenchymal cells isolated from the embryonic chicken limb bud can differentiate into avian brown adipocyte-like cells that exhibit morphological and many biochemical properties of terminally differentiated brown adipocytes. These avian brown adipocyte-like cells could be similar to brown-like adipocytes, so-called beige adipocytes (Ishibashi and Seale 2010), or brite (brown-in-white) adipocytes (Petrovic et al. 2010) observed in mammals though the expression of any UCP homologs has never been examined. The question of whether ‘beige cells expressing avUCP’ could come from white adipose tissue of birds exposed to cold or other inducers remains an unanswered question in the literature. Here, the chicken was used as avian model to clarify if ‘beige cells’ could appear based on in vivo experiments. The potential physiological value of beige adipocytes browning obtained in the avian models can be extrapolated with care, to birds, given that some of the avian physiological characteristic relevant to energy metabolism, such as the lack of glut4 and the advent of avUCP are evidenced with chickens (Raimbault et al. 2001; Newman et al. 2013; Seki et al. 2003).

We applied cold- and T_3_-treatments, which are both effective stimuli to shift WAT into beige fat in mammals (Kajimura et al. 2015; Barbatelli et al. 2010; Matesanz et al. 2017; Lee et al. 2012), to laying-type chickens which have much more tolerance to heat or cold environmental stress than a meat-type chickens (Mujahid et al. 2005; Azad et al. 2010).

Enhancements in gene expression of *avUCP* in the neck subcutaneous adipose tissues of cold-acclimated or T_3_-treated chickens were observed (Fig. 1b), which are in agreement with the results obtained in mice by Guerra et al. (1998), who showed that, after adrenergic signaling stimulation by cold exposure or treatment with a β_3_-adrenergic agonist, increased *UCP1* mRNA levels were accompanied by a corresponding increase in brown adipocytes in white retroperitoneal fat as revealed by the anti-UCP1 antibody staining. In addition to *avUCP*, the expression level of *CIDEA* [a known mammalian marker for both brown and beige adipocytes (Ikeda et al. 2018; Petrovic et al. 2010; Sharp et al. 2012; Wu et al. 2012)], and those of *TBX1*, *TMEM26, CAR4*, and *CPT-1b* [known mammalian beige adipocyte-specific markers (Ikeda et al. 2018; Sharp et al. 2012; Wu et al. 2012; Harms and Seale 2013)], increased in the neck subcutaneous fat of control, T_3_-treated and cold-acclimated group in ascending order. Expression levels of *SLC27A1*, *CD137*, and *EAR2* [known mammalian beige adipocyte-specific markers (Sharp et al. 2012; Wu et al. 2012)] were not changed (Fig. 1c). Those apparent discrepancies between the mammalian results and ours in the gene expression of *SLC27A1* and *CD137* may have arisen from use of different species and type of animals.

The lack of increase in *SLC27A1* in this study may simply be related to bird-specific lipid metabolism, considering that, different from mammals, fat deposition in birds depends on exogenous fatty acid provision much more than de novo synthesis (Griffin et al. 1992). *SLC27A1* may not be a rate-limiting transporter in fatty acids uptake for mitochondrial β-oxidation, which might be orchestrated by different other transporters, *CD36* and *ACS1* beside *SLC27A1*.

Although *CD137* was identified as a marker of beige adipocyte in DNA microarray screens of immortalized cell lines derived from inguinal WAT of a strain of obesity-resistant 129SVE mice (Wu et al. 2012), subsequent studies were unable to validate enrichment of *CD137* in beige adipocytes, or increased *CD137* expression after cold exposure or adrenergic stimulation in mice (Rosenwald et al. 2013; Srivastava et al. 2020). In agreement with those studies, we did not observe increased expression of *CD137* mRNA upon cold- and T_3_-treatments. In these regards, additional studies will be required to clarify the chicken-specific functions of *SLC27A1* and *CD137* related to lipid catabolism and immunoreactivity in beige adipocytes.

On the other hand, in abdominal fat, gene expression of *avUCP* was scarcely detected regardless of either treatment (Fig. 1b), or even if detected, was observed to be very low in control and T3-treated chickens (Fig. 3a). Furthermore, the levels of all marker examined for both brown and beige adipocytes were not increased by either treatment in abdominal fat (Fig. 1c). Therefore, even though the islets of multilocular fat cells emerging within neck subcutaneous fat and abdominal fat following both cold- and T_3_ stimulation are characterized by an increased number of clusters containing small adipocytes (Fig. 2c), these results suggest that beige-like adipocytes may be predominantly present in the neck subcutaneous fat rather than in abdominal fat tissues of chicken exposed to cold- and T_3_-treatment.

Since the observed increase in *avUCP* expression as well as the number of multilocular fat cells within neck subcutaneous fat are indicative of the presence of beige adipocytes, this tissue may have thermogenic capacities to promote energy expenditure. Himms-Hagen et al. (2000) observed that multilocular fat cells were rich in mitochondria and positive for UCP1 by immunohistochemistry in the WAT of rats treated with the β_3_-adrenoceptor agonist CL-316243. We confirmed similar results in chicken by showing the presence of both functional mitochondria and avUCP protein in multilocular fat cells. We experimentally measured the expression of the mitochondrial outer membrane protein, VDAC (Bathori et al. 2006), and the mitochondrial inner membrane protein avUCP in the WAT of T_3_-treated chickens. An increased abundance of avUCP at the protein level and the VDAC protein were found as well as that of *avUCP* mRNA in neck subcutaneous fat tissues (Fig. 3a–c). Based on the immunohistochemical analysis, the neck subcutaneous fat of T_3_-treated chickens presented small clusters that were positive for VDAC, corresponding to multilocular fat cells (Fig. 3d), which shows that the dense clusters of beige-like adipocytes emerge in mitochondria-rich tissue of chickens. Our morphological and immunohistochemical results for beige-like adipose tissue in chickens are consistent with the criteria adopted by Sidossis et al. (2015), namely that the browning of subcutaneous WAT is determined by the presence of multilocular adipocytes, UCP1, and increases in mitochondrial density and respiratory capacity. Therefore, the increased *avUCP* transcript and protein levels within the multilocular fat cells in the neck subcutaneous fat of T_3_-treated chickens are evidences for the presence of beige-like adipocytes. However, this seems not to be the case for abdominal fat because it was devoid of *avUCP*, or exhibited low *avUCP* expression and VDAC protein., where triglycerides must first be also hydrolyzed and some of the resultant fatty acids delivered to the thermogenarating muscles. These depot-specific effects are supported by the differences observed in the proportions of UCP1-immunoreactive adipocytes in the subcutaneous and visceral WAT as reported in 129 Sv mice (Barbatelli et al. 2010); after 10 days’ exposure to 6 °C, brown adipocytes significantly increased in subcutaneous WAT, whereas they were not significantly increased in visceral WAT. Our results suggest that abdominal fat exhibits increased lipolysis probably without increased thermogenesis while neck subcutaneous fat has increases in both lipolysis and thermogenesis.

Is avPGC-1α involved in the upregulation of avUCP in adipose tissue after stimulation? In mammals, the transcriptional regulator gene *PGC-1α* in BAT is required to coordinate the expression of *UCP1* (Lowell and Spiegelman 2000), while expression of *PGC-1α* in WAT is also induced by cold stimulation and dietary supplementation with T_3_, indicative of the role for *PGC-1α* in beige adipocytes of mammals (Barbatelli et al. 2010; Lee et al. 2012). In chickens, upregulation of *avPGC-1α* expression precedes an increase in *avUCP* expression in the mitochondria of skeletal muscle (Ueda et al. 2005). Therefore, *avPGC-1α* may be involved in regulating *avUCP* expression in neck subcutaneous adipose tissues. Results of this work showed a positive correlation between *avUCP* and *avPGC-1α* mRNA levels (Fig. 1d, Fig. S1), suggesting that *avPGC-1α* in neck subcutaneous adipose tissues is important for regulating the expression of mitochondrial *avUCP* in chickens under cold exposure or T_3_ treatment. This hypothesis is supported by the correlated alteration in *UCP* and *PGC-1α* mRNA levels during development of brown adipocytes in retroperitoneal white fat of mice (Xue et al. 2007).

Another important question emerging from the current study is whether avUCP functions as a thermogenic protein in chickens by mediating avUCP-induced proton leak. The functional assessment of beige adipose tissue and UCP1, and whether beige adipocytes generate a sufficient amount of heat that will substantially support thermoregulation remains unresolved in mammals (Keipert and Jastroch 2014). Even if UCP1 activity would directly produce heat, it should be noted that a robust UCP1-independent thermogenic mechanism in beige fat can also involve either enhanced ATP-dependent Ca^2+^ cycling by sarco/endoplasmic reticulum Ca^2+^-ATPase 2b (SERCA2b) and ryanodine receptor 2 (RyR2) (Ikeda et al. 2017), or a futile creatine-driven cycle coupled to mitochondria ATP synthesis (Kazak et al. 2015). Both are noncanonical thermogenic mechanisms through which beige fat controls whole-body energy homeostasis. In birds, although the physiological role of avUCP remains controversial (Emre et al. 2007; Mozo et al. 2005), it was previously reported that cold acclimation (4–6 °C for 10–12 days) induces fatty acid-mediated uncoupling of mitochondrial oxidative phosphorylation in the subsarcolemmal mitochondria isolated from chicken skeletal muscle (Toyomizu et al. 2002) and that the mitochondria from chicken skeletal muscle exposed to cold for 2 d and 2.5 d (4 °C to 6 °C) exhibited uncoupling by both endogenous and exogenous fatty acids (Ueda et al. 2005). Thus, an increased supply of non-esterified fatty acids to cold-acclimated skeletal muscles may contribute to thermogenesis in chickens via avUCP, responsible for uncoupling of mitochondrial oxidative phosphorylation processes (Barre et al. 1986b; Duchamp et al. 1992; Roussel et al. 1998; Wojtczak and Schonfeld 1993). This fatty acids-mediated uncoupling probably happens with even beige-like adipose tissue. However, it cannot be also ruled out that avUCP may play a role in alleviating the overproduction of reactive oxygen species (Abe et al. 2006; Rey et al. 2010) as shown for UCP1 of BAT (Dlasková et al. 2010), resulting from a stimulation of the ATP-consuming process of sarcoplasmic reticulum Ca^2+^ cycling or futile creatine substrate cycling, which could produce heat as described above.

## Perspective and significance

Non-shivering thermogenesis in BAT is induced by cold via thyroid hormones and the sympathetic nervous system, and is an essential mammalian mode of protection against hypothermia, especially in young and hibernating animals (Cannon and Nedergaard 2004). Beige adipocytes detected in animals exposed to cold or other inducers also contribute to thermogenesis. However, birds do not appear to possess BAT, while in vivo emergence of beige fat has not been reported for at least the last two decades. Although previous studies have suggested that thermogenesis in birds in response to cold or thyroid hormones is mediated entirely by skeletal muscle (Newman et al. 2013), skeletal muscle thermogenesis is unlikely sufficient to maintain the body temperature of birds around some regions of the animal due to sparse feathering. Our study on chickens showed that levels of the avUCP protein are correlated with the presence of beige-like adipocytes in response of cold environments, implying that thermogenesis in chickens might be associated with avUCP in beige-like adipocytes as well. Given that some of avian physiological characteristic relevant to energy metabolism were evidenced with chickens, our current results can be used to infer that birds may acquire the capacity for non-shivering thermogenesis by converting some white adipocytes into beige-like adipocytes, thus highlighting the potential physiological value of beige-like adipocytes in birds. Our new finding warrants further investigation.

## Supplementary Information

Below is the link to the electronic supplementary material.

Supplementary file1 (DOCX 3881 KB)

## Data Availability

All data in the figures, image analyses and tests are available from the authors upon reasonable request.

## Abbreviations

UCP: Uncoupling protein
VDAC: Voltage-dependent anion channel
WAT: White adipose tissue
BAT: Brown adipose tissue
CIDEA: Cell death inducing DNA fragmentation factor like effector a
TBX1: T-box transcription factor 1
TMEM26: Transmembrane protein 26
CAR4: Carbonic anhydrase 4
SLC27A1: Solute carrier family 27 member 1
CD137: Cluster of differentiation 36
NR2F6: Nuclear receptor subfamily 2 group F member 6
CPT1b: Carnitine palmitoyltransferase 1b
T_3_: Triiodothyronine
avPGC-1α: Avian peroxisome proliferator-activated receptor γ coactivator-1α
18S: 18S-ribosomal RNA; H&E, hematoxylin and eosin
