# Supplementary material for: In vivo emergence of beige-like fat in chickens as physiological adaptation to cold environments
Source: Amino Acids. 2021 Feb 17;53(3):381–93. doi: 10.1007/s00726-021-02953-5 (PMC7979618; doi:10.1007/s00726-021-02953-5)
Supplement: Supplementary file 1 — Supplementary file1 (DOCX 3881 KB) [file 726_2021_2953_MOESM1_ESM.docx]

***Supporting Information:***

***In vivo* emergence of beige-like fat in chickens as physiological adaptation to cold environments**

**Rina Sotome^1^, Akira Hirasawa^2^, Motoi Kikusato^1,3^, Taku Amo^4^, Kyohei Furukawa^1,3^, Anna Kuriyagawa^1^, Kouichi Watanabe^3,5^, Anne Collin^6^, Hitoshi Shirakawa^3,7^, Ryota Hirakawa^1,3^, Yuta Tanitaka^1^, Hideki Takahashi^3,8^, Guoyao Wu^9^, Tomonori Nochi^3,5^, Tsuyoshi Shimmura^10^, Craig H. Warden^3,11^, Masaaki Toyomizu^1,3^***

^1^ Animal Nutrition, Life Sciences, Graduate School of Agricultural Science, Tohoku University, Sendai, Japan 980-8572

^2^ Department of Genomic Drug Discovery Science, Graduate School of Pharmaceutical Sciences, Kyoto University, Kyoto, Japan 606-8501

^3^ International Education and Research Center for Food and Agricultural Immunology, Graduate School of Agricultural Science, Tohoku University, Sendai, Japan

^4^ Department of Applied Chemistry, National Defense Academy, 1-10-20 Hashirimizu, Yokosuka, Japan 239-8686

^5^ Functional Morphology, Life Sciences, Graduate School of Agricultural Science, Tohoku University, Sendai, Japan 980-8572

^6^ INRAE, Université de Tours, BOA, 37380 Nouzilly, France

^7^ Nutrition, Graduate School of Agricultural Science, Tohoku University, Sendai, Japan 980-8572

^8^ Plant pathology, Life Sciences, Graduate School of Agricultural Science, Tohoku University, Sendai, Japan 980-8572

^9^ Department of Animal Science, Texas A & M University, 2471 TAMU, College Station, Texas, USA 77843-2471

^10^ Graduate School of Agricultural Science, Tokyo University of Agriculture and Technology, Fuchu 183-8509, Japan

^11^ Department of Pediatrics, Section of Neurobiology, Physiology and Behavior and Rowe Program in Genetics, University of California, Davis, CA 95616, USA

Numbers of Pages: 13

Numbers of data: 2

Numbers of Figures: 4

Numbers of Tables: 1

**Table of contents Page**

**Supplement A Uncropped Western blots of Figure 3B and observed band size on Western blot for avUCP S4**

**Supplement B Uncropped Western blots for Figure 3C and membranes stained with Ponceau-S S7**

**Figure S1 Dependency of *avUCP* expression on *avPGC-1α* in neck subcutaneous fat of control and**

**T_3_-treated chickens (n = 11), and effects of T_3_ on mRNA levels of *avPGC-1α*. S9**

**Figure S2 mRNA levels of *avPGC-1α* in abdominal fat of control and T_3_-treated chickens S10**

**Figure S3 VDAC protein content in abdominal fat of control and T_3_-treated chickens S11**

**Figure S4 Immunohistochemical analysis using anti-VDAC antibody for abdominal fat of control**

**and T3-treated chickens S12**

**Table S1 Primer sequences used for gene expression analysis S13**

**Supplement A Uncropped Western blots of Figure 3B and observed band size on Western blot for avUCP**

Uncropped Western blots of Figure 3B are provided below. Considering that the calculated molecular weight of avUCP is 33kDa, the lower band of two observed major bands (~40 kDa and ~30 kDa) is presumed to correspond to avUCP.


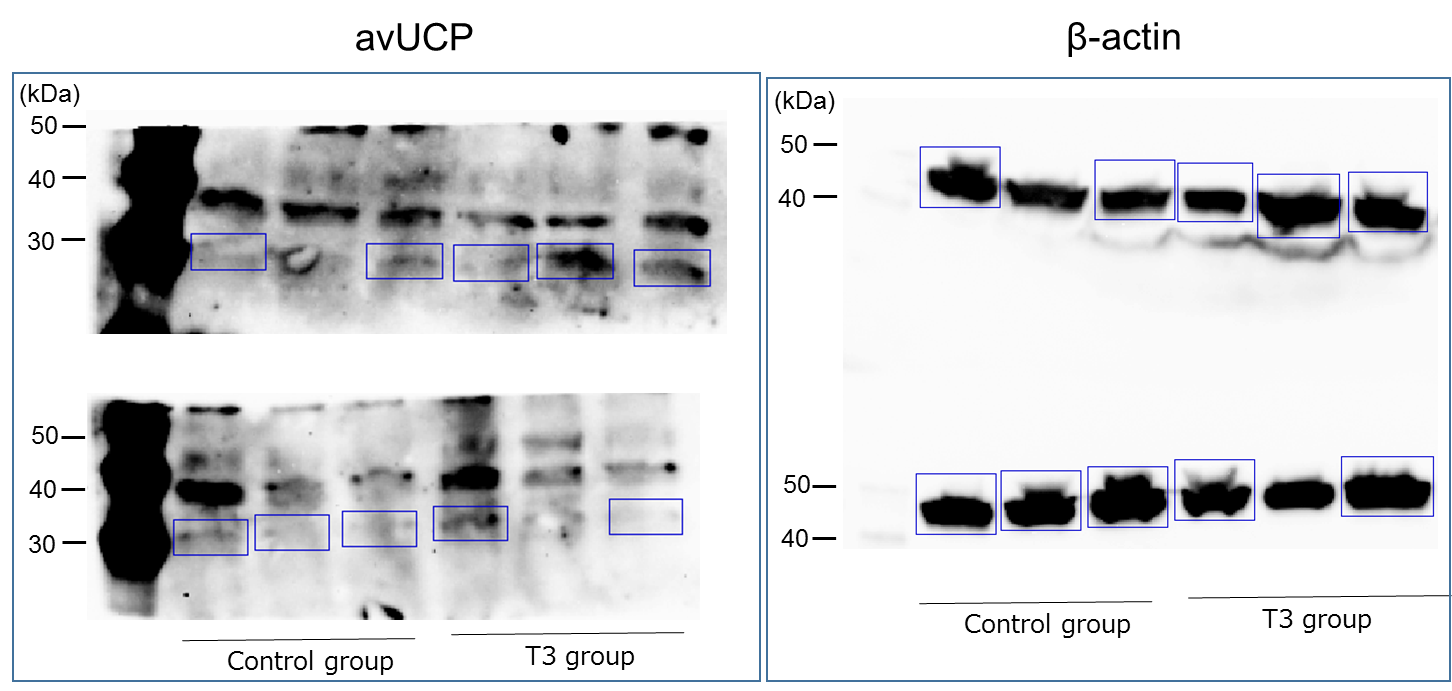


**Uncropped Western blots of Figure 3B**

One experiment was conducted according to the well-known information that the avUCP protein of skeletal muscle is enhanced by fasting in chickens [1] as well as mammals [2]. Male chickens (3-4 week-old) were used for determination of avUCP protein level using Western blotting. Skeletal muscle mitochondria were isolated from the *pectoralis* muscle of 0 and 48h-fasted chickens. As shown below, avUCP protein content from the *pectoralis* muscle, which is detected as band at ~30k Da, significantly increased in 48 h-fasted chickens.


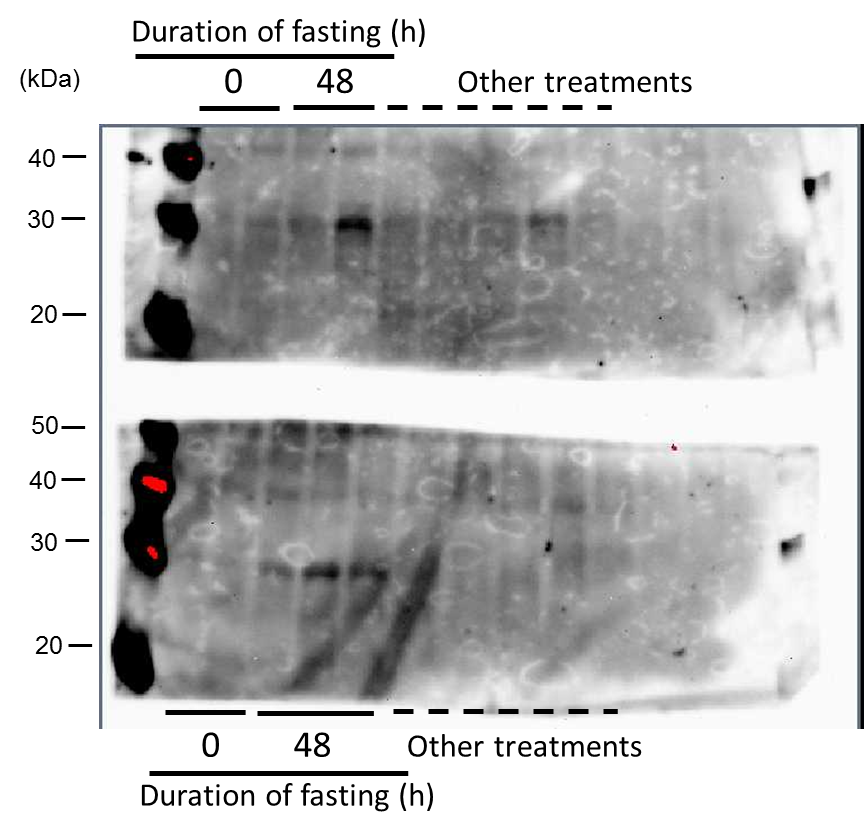


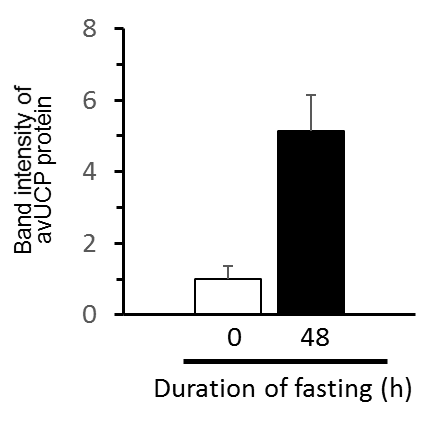


Antibodies used for the detection of avUCP protein and experimental conditions for qualification of avUCP protein using Western blotting were the same as described in the Materials and Methods.

**Observed band size on Western blot for avUCP**

This result confirms that the lower band of the two major bands (~40 kDa and ~30 kDa) in the uncropped Western blots of Figure 3B corresponds to the calculated molecular weight of avUCP, 33kDa.

[1] FEBS letters 580: 4815-4822, 2006

[2] Diabetes 47: 1693-1698. 1998

**Supplement B Uncropped Western blots for Figure 3C and membranes stained with Ponceau-S**

1.
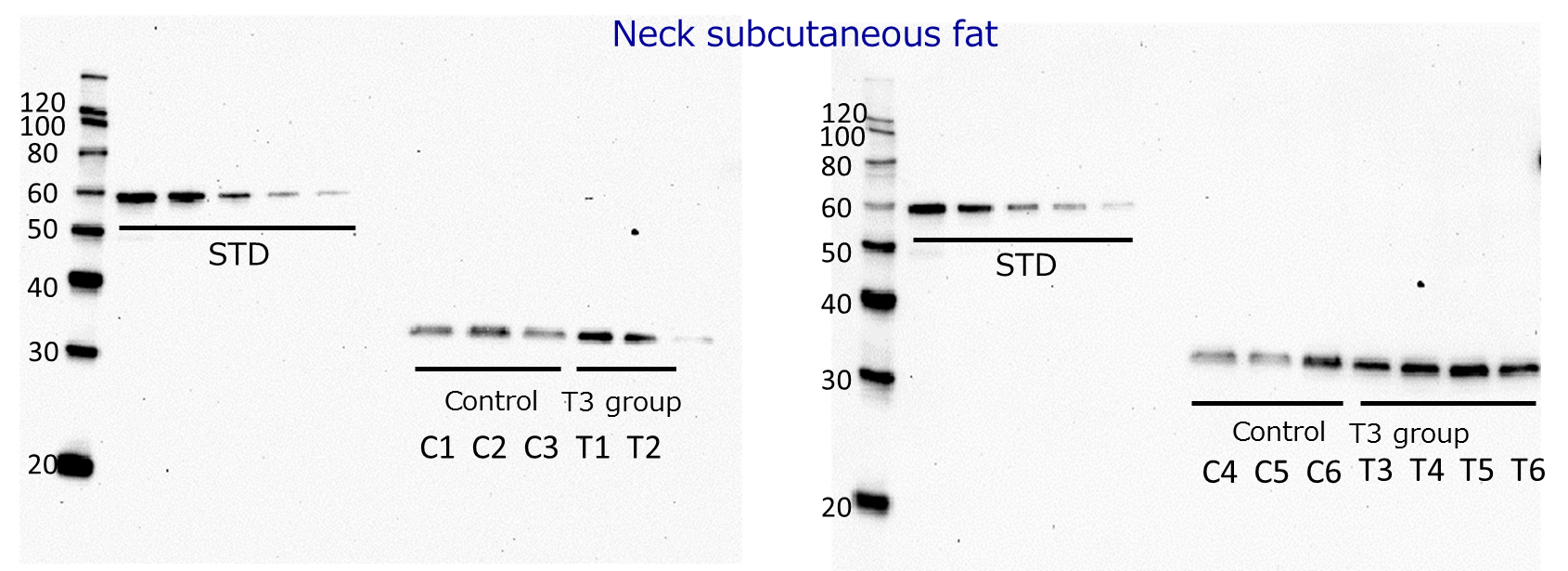
 **Uncropped Western blots for Figure 3C**

The antibody used for VDAC was obtained from https://www.citeab.com/antibodies/758998-ab15895-anti-vdac1-porin-antibody-mitochondrial-l. The peptide antigen sequence was almost identical to the chicken VDAC (97% identity) with a clear single band having an appropriate molecular weight being observed by Western blotting. The VDAC1/Porin protein content was quantiﬁed based on the standard curves obtained from serial dilutions of recombinant human VDAC/Porin (ab132481, Abcam, Cambridge, UK) on the same membrane. The predicted band size of the recombinant human VDAC for standard is 57 kDa including the GST tag N-terminus. (see https://www.abcam.co.jp/recombinant-human-vdac1porin-protein-ab132481.html)

1. **The membranes stained with Ponceau-S of full length-blots for Figure 3C**


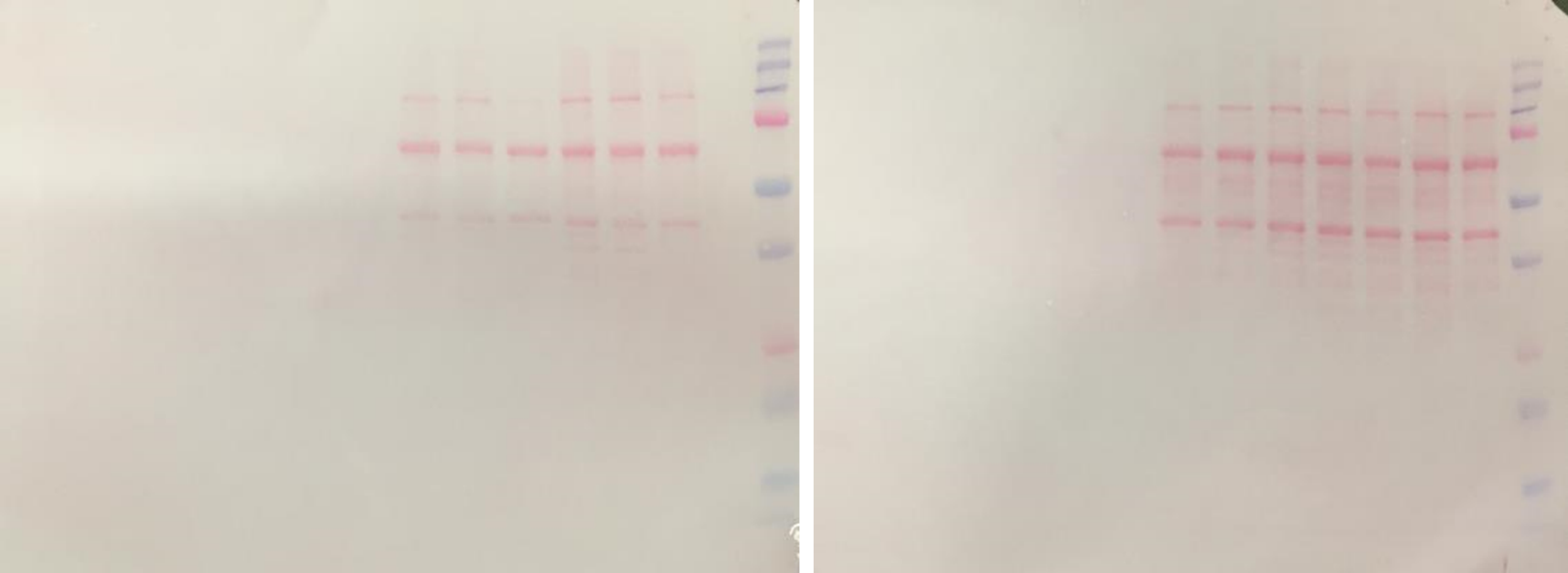


The membranes were stained with Ponceau-S (#SP-4030, Aproscience, Tokushima, Japan) according to the manufacturer’s instructions to confirm similarities of transfer efficiency among the loading western blot samples.

**Figure S1 Dependency of *avUCP* expression on *avPGC-1α* in neck subcutaneous fat of control and T_3_-treated chickens (n = 11), and effects of T_3_ on mRNA levels of *avPGC-1α*.**


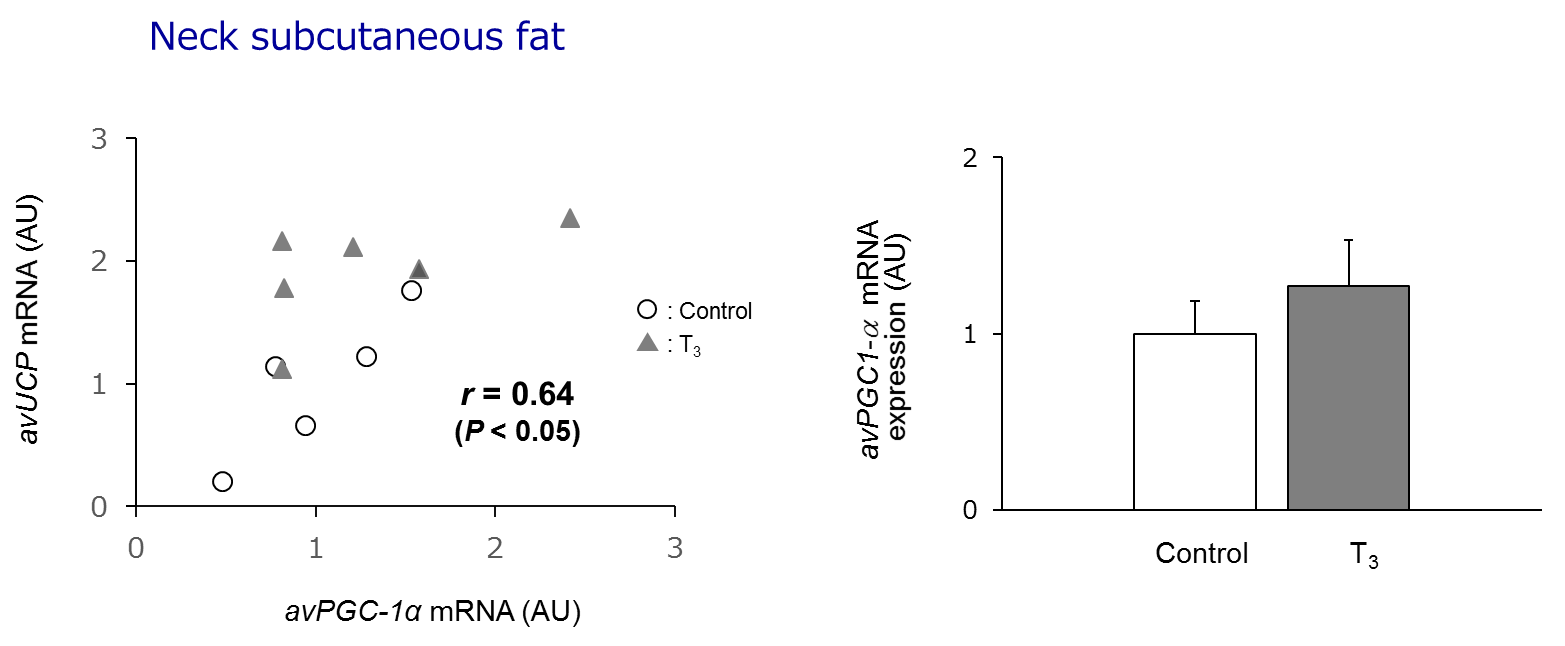


All data for mRNA levels are shown as fold changes relative to control values. Quantitative real-time RT-PCR was used to quantify mRNA levels, and the results were normalized to 18S rRNA levels.

Value represents Pearson correlation coefficients for the relationship between *avUCP* and *avPGC-1α* gene levels.

**Figure S2 mRNA levels of *avPGC-1α* in abdominal fat of control and T_3_-treated chickens**

**
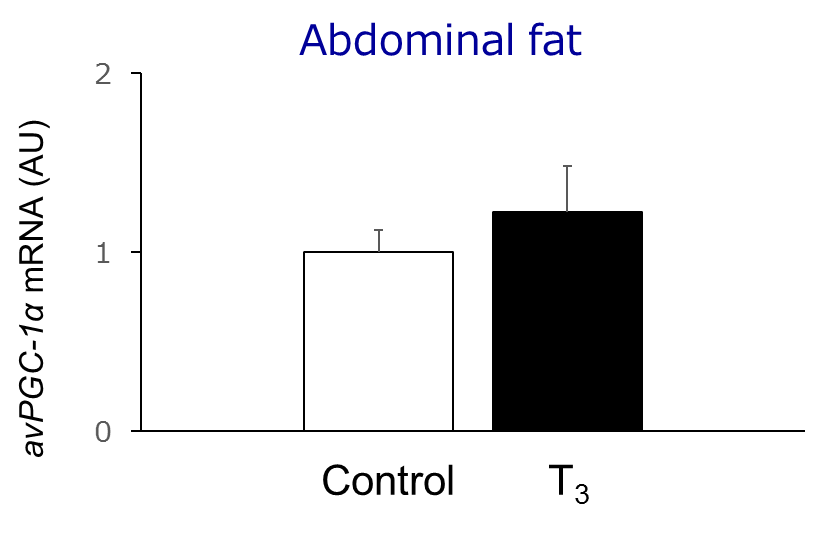
**

All data for mRNA levels are shown as fold changes relative to control values. Quantitative real-time RT-PCR was used to quantify mRNA levels, and the results were normalized to 18S rRNA levels.

**Figure S3 VDAC protein content in abdominal fat of control and T_3_-treated chickens**

**
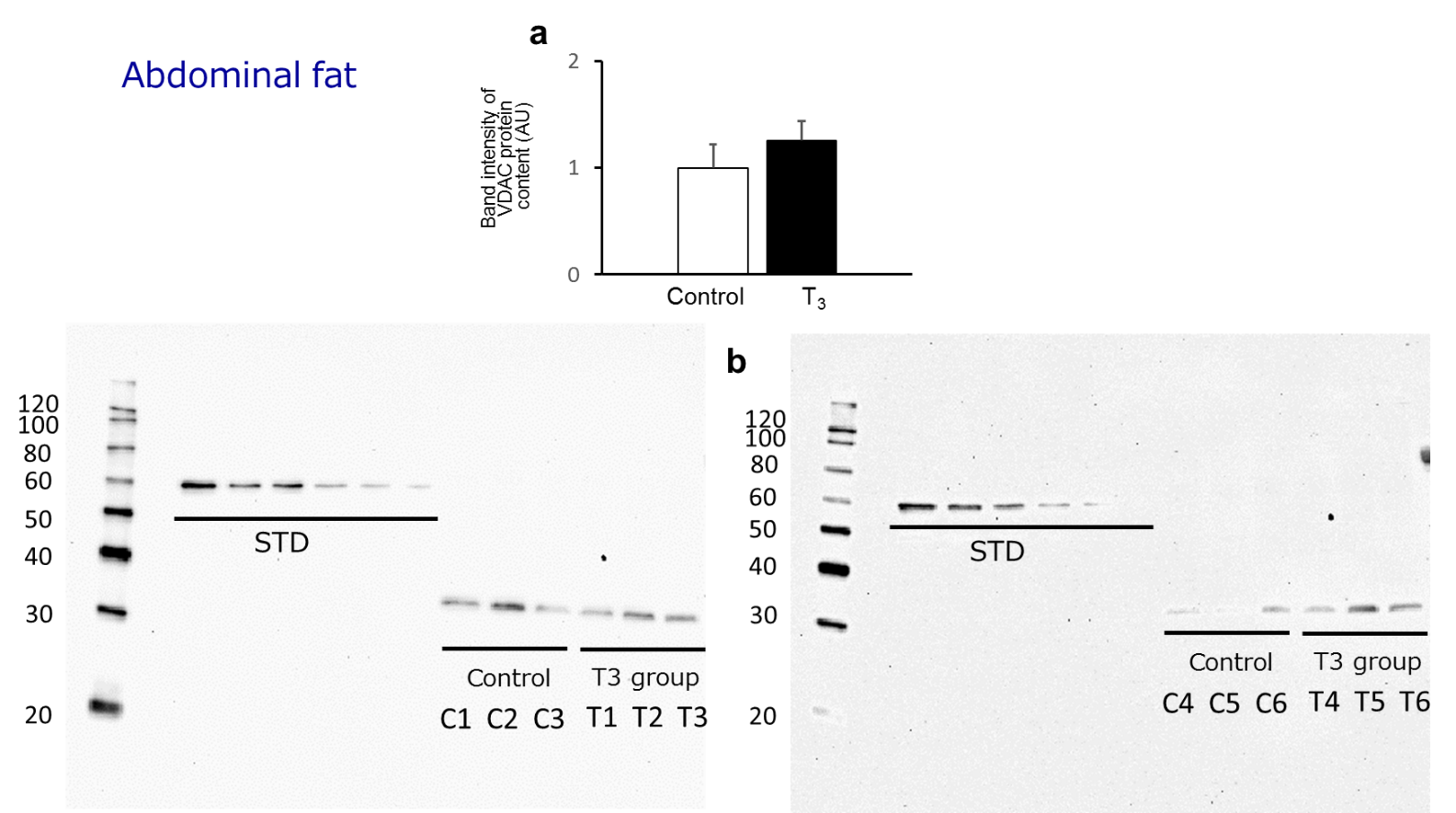
**

VDAC protein content in the abdominal fat of control and T_3_-treated chickens. (a) Band intensities of VDAC shown as semi-quantified by densitometric tracing. (b) VDAC protein levels were assessed by Western blot analysis of tissue protein (10 μg) using an anti-VDAC antibody. Values are means ± S.E., n = 6 chickens in each group. Information on antibody used for VDAC and the predicted band size of the recombinant human VDAC/Porin for standard are given in Supplement B in the Supporting Information.

**Figure S4 Immunohistochemical analysis using anti-VDAC antibody for abdominal fat of control and T3-treated chickens**

**
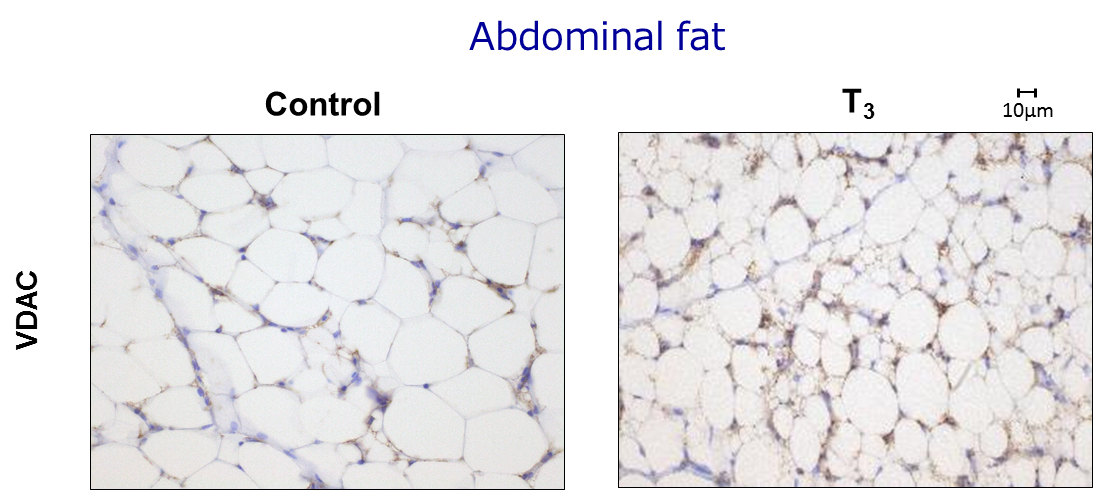
**

**Table S1 Primer sequences used for gene expression analysis**

| Gene | Primer set | | Annealing temp. (°C) | GenBank® accession No. |
| --- | --- | --- | --- | --- |
| *avUCP* | *Sense* | ACTCTGTGAAGCAGCTCTACACC | 66 | AB088685 |
|  | *Antisense* | ATGTACCGCGTCTTCACCACATC |  |  |
| *avPGC-1α* | *Sense* | ATCAGAACAAGCCCTGTGGT | 60 | NM_001006457 |
|  | *Antisense* | GACTCAGGTGTCAATGGAAGTG |  |  |
| *CIDEA* | *Sense* | GCTCGGTGCTGCGATCTTT | 64 | NM_001195123 |
|  | *Antisense* | AGGACCATGAAGTGCGTGTT |  |  |
| *TBX1* | *Sense* | GGACATGGAAGCTATCTCCAGC | 60 | XM_025155713.1 |
|  | *Antisense* | GTGAAAGCTGCAACATCGCAA |  |  |
| *TMEM26* | *Sense* | TCGGACGGCTAAAGTCTTGG | 60 | NM_001199598 |
|  | *Antisense* | GAC AAC TGG TCC CGT GTG AT |  |  |
| *CAR4* | *Sense* | TGCTGTTCCTTGTTCTGTTCTC | 63 | XM_415893.6 |
|  | *Antisense* | ATGCCAGTGTCGAGGGTCTT |  |  |
| *SLC27A1* | *Sense* | TACGGAGCCACCGAGTGCAACT | 57 | NM_001039602.2 |
|  | *Antisense* | CGCACAGCCCTCTGGAATCACG |  |  |
| *CD137* | *Sense* | AACGGGTCAGGTAAAGGGTG | 57 | XM_015297003.2 |
|  | *Antisense* | CTTTGGAATTCAGGTTTCTGTAGT |  |  |
| *EAR2* | *Sense* | CAGTACTGCCGCCTGAAGAAG | 65 | XM_015300096.2 |
|  | *Antisense* | GATACCGGCTGCCCGTTGAA |  |  |
| *CPT1b* | *Sense* | GATTTCTGCTGCTTCCAATTCG | 63 | DQ314726.1 |
|  | *Antisense* | TGCAGCGCGATCTGAATG |  |  |
| *18S* | *Sense* | TAGATAACCTCGAGCCGATCG | 63 | AF173612 |
|  | *Antisense* | GACTTGCCCTCCAATGGATCC |  |  |

*Abbreviations:* *avUCP*, avian uncoupling protein; *avPGC-1α*, avian peroxisome proliferator-activated receptor gamma coactivator 1 alpha; *CIDEA*, cell death-inducing DNA fragmentation factor, α-subunit-like effector A; *TBX1*, T-box protein 1; *TMEM26*, transmembrane protein 26; *CAR4*, carbonic anhydrase 4; *SLC27A1*, solute carrier family 27 member 1; *CD137*, cluster of differentiation 36; *EAR2*, NR2F6: nuclear receptor subfamily 2 group F member 6; *CPT1b*, carnitine palmitoyltransferase 1b and *18S*, 18S-ribosomal RNA.
